# RIPK3 Expression in Fibroblasts in an in vivo and in vitro Skin Wound Model: A Controversial Result

**DOI:** 10.32607/actanaturae.25452

**Published:** 2023

**Authors:** I. S. Izumov, M. S. Shitova, M. S. Sabirov, S. A. Sheleg, O. L. Cherkashina, E. P. Kalabusheva, E. A. Vorotelyak, E. I. Morgun

**Affiliations:** Koltzov Institute of Developmental Biology of Russian Academy of Sciences, Moscow, 119334 Russian Federation; M.V. Lomonosov Moscow State University, Moscow, 119234 Russian Federation

**Keywords:** scarring, keloid, skin, fibroblasts, cell culture, RIPK3

## Abstract

One of the major problems of regenerative medicine is the development of hypertrophic scars and keloids. The protein kinase RIPK3 is involved in necroptosis; however, recent evidence indicates that it also has non-canonical functions, including its involvement in the development of renal fibrosis. The aim of our work was to study the expression of RIPK3 in mouse and human skin models of fibrotic processes. A subpopulation of RIPK3+Vim+ cells was found in both human keloid and a mouse wound, with the cell number being significantly greater in the mouse wound bed compared to healthy skin. Real-time polymerase chain reaction (RT-PCR) detected expression of the *Ripk3 *and fibroblast biomarkers *Acta2*, *Fap*, *Col1a1*, and* Fn1 *in the cells isolated from the wound bed, indicating that RIPK3 can be expressed by wound bed fibroblasts. An analysis of the human fibroblasts stained with anti-RIPK3 antibodies demonstrated an increase in the fluorescence intensity in the presence of lipopolysaccharide (LPS) at concentrations of 5, 10, 25, 50, and 100 ng/ml and TGF-β at concentrations of 0.1, 1, 2, and 5 ng/ml compared to the control. At the same time, the expression levels of *RIPK3 *and fibroblast activation markers in the presence of TGF-β and LPS did not differ significantly from the control. It is possible that RIPK3 expression in wound fibroblasts is not directly associated with fibrotic processes, and that kinase plays a different, yet unknown role in wound healing. KEYWORDS scarring, keloid, skin, fibroblasts, cell culture, RIPK3.

## INTRODUCTION

Disorders of skin wound healing is a major medical problem. These disorders include pathologies associated with fibrotic processes, which are caused by enhanced proliferation of fibroblasts and excessive synthesis of the extracellular matrix (ECM), leading to hypertrophic and keloid scarring. There are approaches to the treatment of skin wounds [1]; however, the problem of regeneration anomalies, such as fibrosis, remains unresolved.

Protein kinase RIPK3 (Receptor-interacting serine/ threonine-protein kinase 3) is an important member of necroptosis, the process of programmed cell death with morphological signs of necrosis. Protein kinases RIPK3 and RIPK1 are known to transmit a signal from receptors such as TNFR, FasR, TRAILR, TLR3, TLR4, and INFAR1 to MLKL, resulting in cell death [2, 3].

RIPK3 not only participates in necroptosis but also possesses non-canonical functions: it is involved in apoptosis and inflammation. RIPK3 promotes cytokine production in dendritic cells [4]. Recently, data has appeared on a possible involvement of RIPK3 in the development of fibrotic processes in kidneys and lungs [5, 6]. Previous experiments performed in our laboratory have demonstrated RIPK3 expression in mouse and human skin [7]. In this regard, the aim of our work is to study RIPK3 expression in mouse and human skin models of fibrotic processes.

## EXPERIMENTAL

**Biological sample**

Thirty male C57Bl/6 mice were used in the study. Mice were housed at +23°C, with unlimited access to drinking water and food (according to GOST No. 33215-2014). All manipulations with the animals were carried out under general anesthesia, in accordance with “Regulations for studies using experimental animals” (Russia, 2010) and “International Guiding Principles (Ethical Codes) for Biomedical Research Involving Animals” (CIOMS and ICLAS, 2012), with the approval of the Bioethics Commission of the Institute of Developmental Biology of the Russian Academy of Sciences (protocols No. 51 of 09.09.2021 and No. 62 of 01.09.2022) and in strict compliance with the ethical principles established by the European Convention for the Protection of Vertebrate Animals used for Experimental and Other Scientific Purposes (Strasbourg, 2006).

In addition to the biological mouse sample, we used a keloid tissue sample and a normal human breast skin sample. Fragments of human skin were obtained by surgery, with the voluntary informed consent of the patient; experiments using cell cultures were carried out with the approval of the Bioethics Commission of the Institute of Developmental Biology of the Russian Academy of Sciences.

**Cell isolation from wounds and intact mouse dermis**

Biological samples were washed in Hank’s Balanced Salt Solution supplemented with amphotericin B solution (Sintez OAO, Russia) and a gentamicin sulfate solution (BioPharmGarant, Russia). Tissues were minced and placed in 0.2% dispase solution (Gibco, catalog No. 17105-041). The samples were incubated in a thermal cycler at +37°C for 30 min. The epidermis was removed from tissue fragments in sterile conditions. The wound specimen was placed in a 0.2% collagenase I (Worthington Biochemical, catalog No. LS004197) and a IV solution (Gibco, catalog No. 1704-019). The skin was placed in a 0.2% collagenase IV solution (Gibco, catalog No. 1704-019). The resulting solution was centrifuged at +4°C and washed thrice with a sterile ice-cold DPBS solution; the sediment was then pipetted.

**Mouse cell cultures**

A suspension of cells isolated from normal mouse dermis was filtered through a strainer with a pore diameter of 100 µm. The cells isolated from the wound bed and normal dermis were resuspended in DMEM and DMEM Advanced, respectively. Both media were supplemented with 10% fetal bovine serum, 1% glutamine, and 1% penicillin-streptomycin. Next, the cells were seeded in a 96-well plate. RNA was isolated from confluent cells.

**Human cell cultures**

Human fibrobalsts were provided by the “Cell culture collection for biotechnological and biomedical research (general biological and biomedical areas)” center of the Institute of Developmental Biology n.a. N. K. Koltsov of the Russian Academy of Sciences.

Human fibroblasts from three different donors were cultured in 6-well plates containing a DMEM medium (PanEco) with 10% fetal calf serum, 1% glutamine, and 1% penicillin-streptomycin at 3 × 10^5^ cells per well. A total of 24 h after cell seeding, the cell media was substituted with a Opti-MEM medium containing 1% fetal bovine serum [5]. After 60 min, the medium was changed to either a medium containing TGF-β at concentrations of 0.1, 1, 2, 5, and 10 ng/ml [5], lipopolysaccharide (LPS) at concentrations of 1, 5, 10, 25, 50, and 100 ng/ml [8], or a mixture of TGF-β (10 ng/ml) and LPS (100 ng/ml). After 24 h, the cells were fixed and stained with anti-RIPK3 antibodies using the standard laboratory protocol. The experiment was repeated with the exception that TGF-β was added at concentrations of 1 and 10 ng/ml, and LPS was added at concentrations of 10 and 100 ng/ml. Total RNA was isolated after 24 h using columns.

**Mouse skin wound model**

We used the approach presented in [9], which utilized a large (square wound, 1 cm^2^ in area) and a small mouse wound (round wound with a diameter of 4 mm) model. We needed to simulate a small wound. However, it is impossible to isolate fibroblasts at the proliferation stage from a wound with a diameter of 4 mm due to its small size. For this reason, we used a wound with a diameter of 8 mm instead.

The mouse was anesthetized by intraperitoneal administration of Avertin. Veet depilatory cream (France) was used to remove hair in the surgical area. Five circles with a diameter of 8 mm were applied to the mouse back using a stencil; the tissue was excised within the boundaries of the applied circles. The resulting wounds were covered with a plaster (Tegadermtm). The mice were removed from the experiment on day 10 after surgery. Normal back skin of mice was used as a biological control.

**Immunofluorescence staining**

Skin wound specimens on slides and cell cultures in plastic plates were fixed using a 4% PFA solution for 10 min and then washed in phosphate-buffered saline (PBS, three times for 5 min each). The samples were then coated with a blocking solution (5% donkey serum and 1% Triton in PBS) and incubated for 30 min in a humidified chamber at room temperature. The blocking solution was removed, and the primary antibody solution was added. The samples were incubated in a humid chamber at +4°C for at least 12 h.

The samples were washed in PBS, coated with a solution of secondary antibodies, and incubated in a humid chamber at room temperature for 1 h. The nuclei were counterstained with DAPI and mounted with a BrightMount/Plus medium (Abcam, UK).

We used primary antibodies to RIPK3 (Sigma, catalog No. HPA055087, dilution 1 : 500) and Vimentin (Abcam, catalog No. ab24525, dilution 1 : 500) and secondary antibodies AlexaFluor 488 (Abcam, Ab150173, dilution 1 : 500), AlexaFluor 594 (A21207, Invitrogen, dilution 1 : 500), and AlexaFluor 660 (A21074, Invitrogen, dilution 1 : 500). A lymph node was used as a positive control for the antibodies to RIPK3. Fibroblasts were used as a positive control for antibodies to Vimentin. The samples not stained with primary antibodies were used as a negative control.

**Fluorescence microscopy**

A Leica DMI6000 microscope was used for fluorescence microscopy and visualization of the preparations stained with antibodies. Photographs were processed and analyzed using the BZ-II Analyzer (Keyence), LAS X (Leica), ImageJ (FiJi), and STATISTICA (StatSoft) software.

**RNA isolation, reverse transcription, PCR followed by gel electrophoresis and RT-PCR**

RNA was isolated from the cells using columns (Biolabmix and Zymo Research) according to the manufacturer’s instructions (USA, Russia). The samples were treated with DNase (ThermoFisher and Zymo Research); cDNA was synthesized using the MMLV RT kit (Eurogen) with an oligo(dT) primer according to the manufacturer’s protocol. Real-time PCR was performed using the qPCRmix-HS SYBR PCR mixture (Evrogen), according to the manufacturer’s instructions on a LightCycler 96 (Roche, Switzerland). Conventional PCR was carried out using the ScreenMix PCR mixture (Evrogen), according to the manufacturer’s instructions on a T100 Thermal Cycler (Bio-Rad, USA). Horizontal gel electrophoresis was performed in a 2% agarose gel. The results were visualized on a ChemiDoc XRS+ System (Bio-Rad).

Primers were selected using PrimerBlast and PrimerSelect (*Table 1*). Gene expression levels were normalized to those of the housekeeping genes: beta- actin (*Actb*) and glyceraldehyde-3-phosphate dehydrogenase (*GAPDH*) in mouse and human samples, respectively.

**Table 1:** Nucleotide sequences of PCR primers

Primer	Forward primer sequence	Reverse primer sequence
hu FN1	GCACCACCCCAGACATTACT	CGGGACTCAGGTTATCAAAAGTG
hu FAP	ATGGGCTGGTGGATTCTTTGT	ATGTTTGTAGCCATCCTTGTCACT
hu COL1A1	CCCCTGGAAAGAATGGAGATGA	CAAACCACTGAAACCTCTGTGTC
hu GAPDH	GAAGGTCGGAGTCAACGGATTT	TTCTCAGCCTTGACGGTGC
hu RIPK3	ATGCTGCTGTCTCCACGGTAA	AAAGCCATCCATTTCTGTCCCTC
mo Actb	ACCCGCCACCAGTTCG	AGCATCGTCGCCCGC
mo Acta2	CATTGGGATGGAGTCAGCGG	GACAGGACGTTGTTAGCATAGAGA
mo Acta2	CCCTGAAGAGCATCCGACAC	CAGAGTCCAGCACAATACCAGT
mo Fn1	GAGGAAGAAGACAGGACAGGAA	GTCAGAGTCGCACTGGTAGAA
mo Fap	AAGAAGCTCAAAGACGGGGG	TGCAAGGACCACCATACACTT
mo Ripk3	ACACGGCACTCCTTGGTATC	CTTGAGGCAGTAGTTCTTGGTG
mo Col1a1	TGACTGGAAGAGCGGAGAGTA	GGCTGAGTAGGGAACACACA

**Evaluation of the fluorescence intensity of the stained human fibroblast cells**

The average fluorescence intensity was measured using the ImageJ software (FiJi). For a comparative analysis of the fluorescence intensity, same exposure of different samples of fibroblasts stained with the fluorescent antibodies was used. Measurements were taken at 30 points of 3–5 fields of view for the control and experimental groups. The results were analyzed using GraphPad Prism 8 (USA).

**Analysis of *RIPK3 *expression using RNA-seq data**

*Data collection. *Three data sets were extracted from the NCBI GEO database (https://www.ncbi.nlm.nih. gov/geo/). The GSE113619 data set contains bulk RNA sequencing data for 27 samples of normal human skin (control) and 37 samples of keloid-prone skin, with biological replicates taken into account [10]. The GSE130973 data set includes RNA sequencing data on individual cells from five normal human skin samples [11]. The GSE163973 data set contains RNA sequencing data on individual cells from three human keloid scar samples [12].

*Analysis of differential gene expression. *Differential gene expression was analyzed using bulk RNA sequencing data and the EdgeR package (R version) [13].

*Processing and analysis of individual cell RNA sequencing data. *The Seurat v4.1.1 R package was used for data processing and analysis [14]. Fibroblasts from the datasets GSE113619 and GSE163973 were integrated using canonical correlation analysis (CCA). Data dimensionality reduction was performed using principal component analysis (PCA) of 3,000 highly variable genes (HVGs). The search for the nearest neighbors was performed using the *FindNeighbors* function for the first 30 PC’s. Clustering was performed using the *FindClusters *function with the resolution parameter = 0.1.

**Statistical analysis**

The obtained data were analyzed using the Excel and GraphPad Prism 8 software (USA). Kruskal–Wallis one-way analysis of variance was used to compare multiple groups. The Mann–Whitney U test was used to compare two groups. Data were considered statistically significant at *P* < 0.05.

## RESULTS AND DISCUSSION

**RIPK3 expression in the scar tissue and normal human skin**

**Fig. 1:**
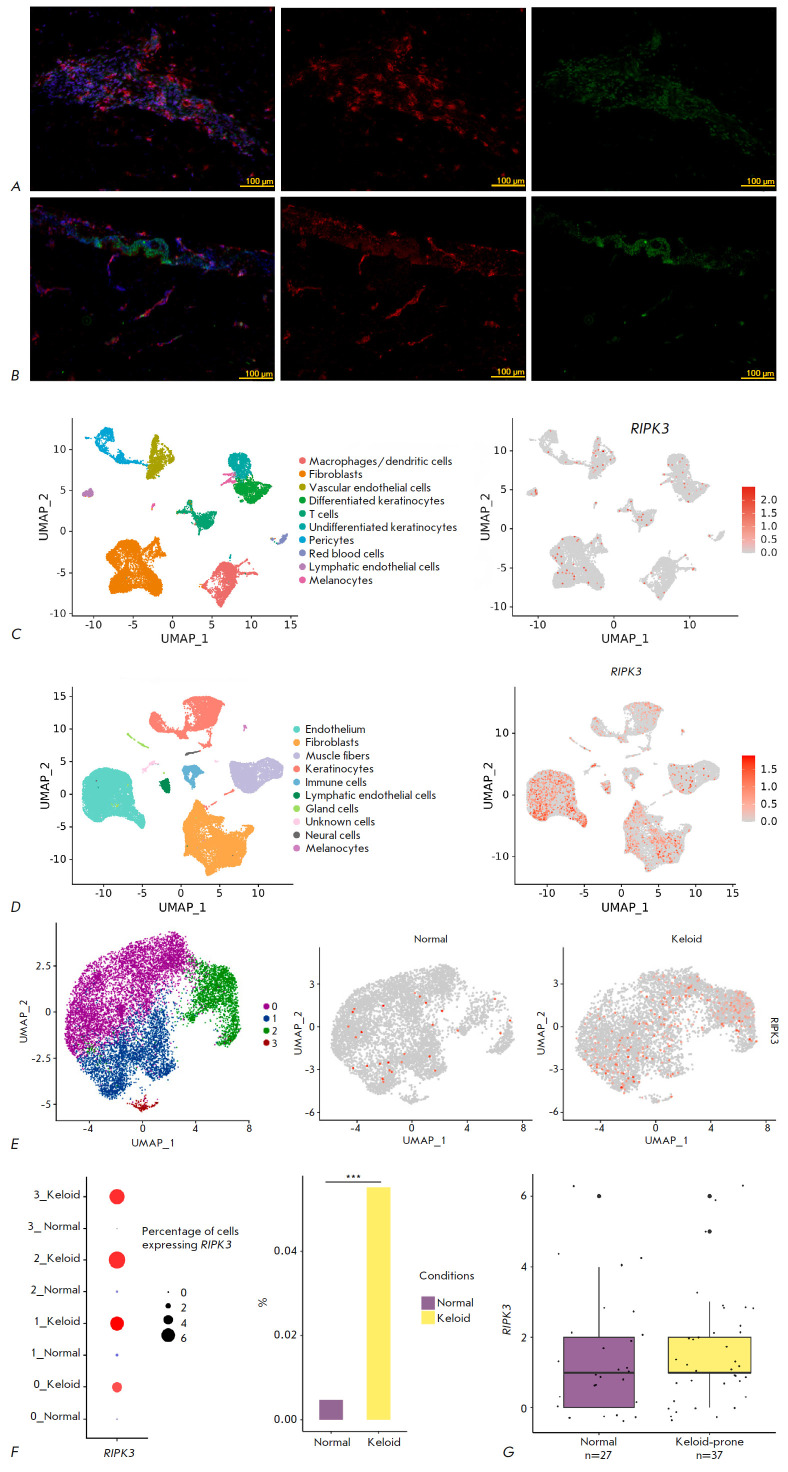
Patterns of RIPK3 expression in human skin. Micropreparations of human keloid scar (*A*) and normal dermis (*B*), immunohistochemical staining with antibodies to Vim (red) and RIPK3 (green), nuclei stained with DAPI, 20× magnification. The UMAP plot of cell clusters with annotations for normal skin samples (left) and the distribution of *RIPK3*+ cells in these data (right) (*C*). The UMAP plot of cell clusters with annotations for normal scar and keloid scar samples (left) and the distribution of *RIPK3*+ cells in these data (right) (*D*). The UMAP plot for cell clusters in fibroblasts from normal skin and keloid scars (left) and the distribution of *RIPK3*+ cells in these data (right) (*E*). The percentage of *RIPK3*+ cells in fibroblasts from normal skin and keloid scars among the four obtained cell clusters (right) and the comparison of the proportions of *RIPK3*+ cells in all fibroblasts from normal skin and keloid scars (left), *** – *P*-value < 2.2e-16 (Fisher’s exact test) (*F*). The distribution of *RIPK3 *raw gene counts in bulk RNA sequencing data on normal and keloid-prone human skin samples (*G*)

Immunofluorescence staining of human keloid showed RIPK3 expression in multiple Vimentin+ cells (*Fig. 1A*). Individual RIPK3+ cells were found in the dermis of normal skin (*Fig. 1B*).

In order to assess a change in the *RIPK3 *expression in keloid scar fibroblasts *in vivo*, we analyzed the RNA sequencing data for human skin samples. We first used the bulk RNA sequencing data obtained by Onoufriadis et al. (GSE113619) in order to determine whether *RIPK3 *belongs to differentially expressed genes (DEGs), compared to normal and keloid- prone skin [10]. The data set included 27 normal skin samples and 37 skin samples from keloid-prone individuals genetically susceptible to form keloids. A comparison of gene expression in normal and keloid- prone skin showed that *RIPK3 *is not a DEG (*logFC = -0.07619307, Padjusted = 1*). *Figure 1G* shows that the distribution of the gene counts in normal skin (light purple range diagram) does not differ from that of the skin in individuals with hereditary susceptibility to form keloids (light golden range diagram). The median count distribution is 1 in both cases.

The low level of *RIPK3 *expression demonstrated in bulk RNA sequencing can potentially be due to the presence of a minor, specific cell population with an active gene. For this reason, we analyzed the results of the sequencing of RNA from individual cells of normal skin and keloid scar. Data on normal skin samples was used from the study by Solé-Boldo et al. (GSE130973) for a visual assessment of *RIPK3* expression in different cell types [11]. In normal skin,* RIPK3 *expression, i.e. *RIPK3+ *cells, was detected at an insignificant level (*Fig. 1C*). Sequencing data for RNA from individual cells of the keloid scar were used from the study by Deng et al. (GSE163973) [12]. Visual evaluation of *RIPK3+ *cell representation demonstrates a significant number of these cells among endothelial cells and the fibroblasts of the keloid scar (*Fig. 1D*). The analyzed data were consolidated and integrated in order to perform a comparative analysis of fibroblasts from healthy skin and keloid scar. The object contained 11,710 cells. Of them, 5,948 and 5,762 cells were normal skin fibroblasts and keloid scar fibroblasts, respectively. We obtained four clusters of fibroblast cells and, similar to the study by Solé-Boldo et al., assessed the distribution of *RIPK3+ *cells between the clusters. As previously demonstrated using data sets containing all skin cell types (*Fig. 1C,D*), the number of *RIPK3+* cells is increased among the fibroblasts of the keloid scar (*Fig. 1E*). Moreover, *RIPK3+ *fibroblasts do not form a separate cluster but instead are distributed randomly. We further compared genes with differential expression in normal skin and keloid scar cells. Similar to the results of bulk RNA sequencing (*Fig. 1G*), *RIPK3 *cannot be considered a DEG, whose expression differs between normal skin and keloid scar fibroblasts. In addition, due to the small number of cells expressing the gene, it was not included in the analysis.

Nevertheless, we see that the percentage of* RIPK3+ *cells in keloid scar fibroblasts is significantly greater than that in normal skin fibroblasts among all cell clusters (*Fig. 1F*, on the left). The difference in the number of *RIPK3+ *fibroblasts (28 out of 5,948 for normal skin cells and 318 out of 5,762 for keloid cells) is statistically significant (Fisher’s exact test,* P*-value < 0.001) (*Fig. 1F*, right). Thus, *RIPK3 *expression in keloid scar fibroblasts is not elevated in* RIPK3+ *cells and corresponds to a physiological level similar to that in normal skin fibroblasts. Moreover, the significant (more than 10-fold) increase in the number of *RIPK3*-expressing cells may be associated with the transition of fibroblasts to an activated state.

**RIPK3 expression in wound and normal mouse tissue**

Modeling of the fibrotic processes in the skin of laboratory mice does not imply a complete transfer of the processes that take place in the human body to the mouse due to the significant morphological and functional differences in the skin structure between mice and humans [15]. For instance, mice are characterized by the presence of the *panniculus carnosus* muscle, which causes rapid wound regeneration by contraction, as well as wound-induced hair neogenesis, which is not characteristic of human skin. However, there exist papers on the study of fibrotic processes in mice. According to Lim et al. and Ito et al., processes occurring in small and large wounds are accompanied by the activation of different signaling pathways and, therefore, have different outcomes [9, 16]. In the study by Lim et al., large wound (≥1cm2) regeneration was accompanied by *Shh *upregulation resulting in wound-induced hair neogenesis in the wound bed and further complete structural and functional skin regeneration. During the healing of small wounds, an increase in *Shh *expression did not occur and, as a result, wound-induced hair neogenesis was not observed. Instead, regeneration outcome in fibrosis [9]. For this reason, we used the small wound mouse model. Considering that excessive scarring can occur due to an enhanced proliferation phase [17], and that the scar itself morphologically and functionally resembles a wound at the proliferation stage, we determined the time point in the regeneration of a mouse wound when it is at the proliferation stage: 10 days after wounding. At this time point, we observed wound closure with hyperproliferative epidermis, granulation tissue with a predominance of the cellular component over fibers, and the absence of hair follicles in mouse wound specimens, which can be considered an immature scar.

**Fig. 2:**
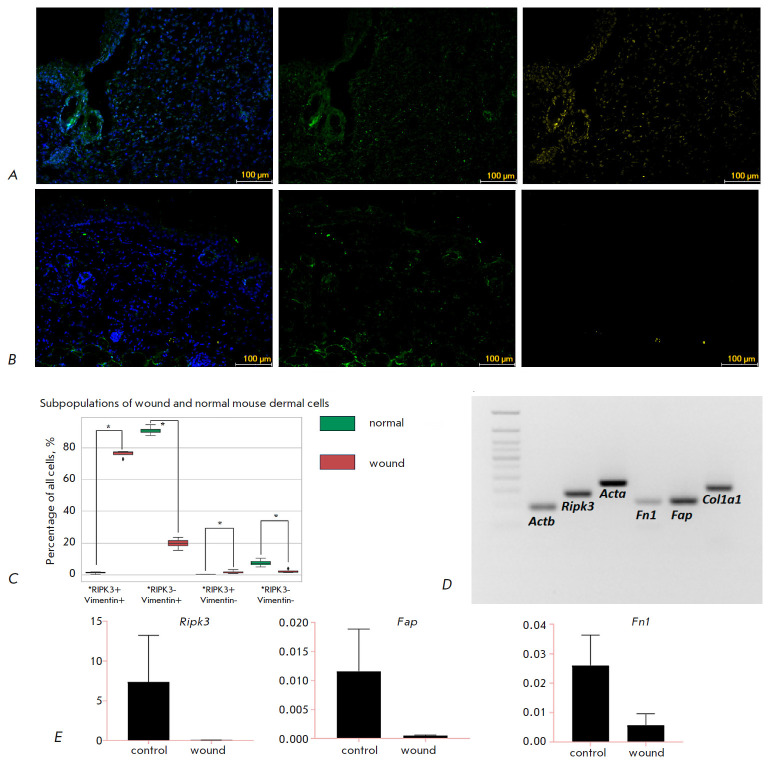
RIPK3 expression patterns in mouse skin. Micropreparations of a wound at the proliferation stage (*A*) and normal mouse skin (*B*), immunohistochemical staining with antibodies to Vim (green) and RIPK3 (yellow), nuclei stained with DAPI, 20× magnification, scale bars 100 μm. (*C*) − Statistical analysis of cell subpopulations in normal mouse dermis and wound bed, *P < 0.05 (Mann–Whitney U-test). Expression of ECM and *Ripk3 *synthesis markers in mouse wound bed cells, PCR followed by gel electrophoresis (*D*); in cultured wound bed cells and intact dermis, RT-PCR, *P *> 0.05 (Mann–Whitney U test, gene expression data are presented as average values with a spread in the form of an average error) (*E*)

An immunofluorescent analysis confirmed the presence of RIPK3+Vim+, RIPK3-Vim+, RIPK3+Vim-, and RIPK3-Vim- cells in the mouse wound bed on regeneration day 10 and in normal skin (*Fig. 2A*). A subpopulation of RIPK3+Vim+ cells prevailed in the wound; the number of RIPK3+Vim+ cells was significantly greater in the wound bed compared to normal skin (*Fig. 2A,C*). The RIPK3-Vim+ cell subpopulation dominated in normal dermis; the number of cells was greater than that in the wound (*Fig. 2B,C*). This result indicates that there was a significantly greater number of RIPK3+ mesenchymal cells in the wound compared to normal dermis. However, not only fibroblasts but also endothelial cells and some inflammatory cells express vimentin. PCR followed by gel electrophoresis of primary cells isolated from the mouse wound bed showed expression of the markers of ECM synthesis and myofibroblast formation; i.e., the processes involved in fibrosis: *Acta2, Fap, Col1a1*, and *Fn1*, as well as *Ripk3* (*Fig. 2D*). In addition, the cells were defined morphologically as fibroblasts. Based on the obtained results, we concluded that the RIPK3+ cells of the mouse wound bed are fibroblasts. Nevertheless, RT-PCR did not show reliable differences in the expression of *Ripk3, Fap, *and *Fn1 *between cultured wound bed cells and the cells isolated from normal dermis; this can be due to a change in the fibroblast phenotype during culture in plastic wells (*Fig. 2E*). It is possible that introduction of normal dermis fibroblasts in the cell culture and their attachment to the plastic surface leads to their *de novo *activation. By that time, granulation tissue fibroblasts are already activated and continue to actively proliferate in the culture, which results in a decrease in the expression of the corresponding genes.

**RIPK3 expression in human dermal fibroblasts in the presence of TGF-β1 and LPS in an *in vitro *model**

According to the data by Imamura, TGF-β causes a dose-dependent increase in RIPK3 expression in NIH 3T3 mouse embryo fibroblasts [5]. It was also shown that, after fibroblast exposure to TGF-β1, RIPK3 can activate the serin/threonine protein kinase AKT. In turn, AKT phosphorylates the ATP citrate lyase ACL, which is involved in fibroblast activation [18, 19, 20].

Another mechanism of RIPK3-mediated regulation of fibrotic processes is possible. The study by Guo et al. suggests a role for TLR4/NF-κB signaling in fibroblast activation, leading to the development of uterine fibroids. LPS induced the expression of collagen type I, TGF-β, and FAP in CD90+ fibroblasts [8]. LPS is also known to activate RIPK3 expression. Thus, we can assume the involvement of RIPK3 in LPSinduced activation of the TLR4/NF-κB signaling pathway in fibroblasts [21].

**Fig. 3:**
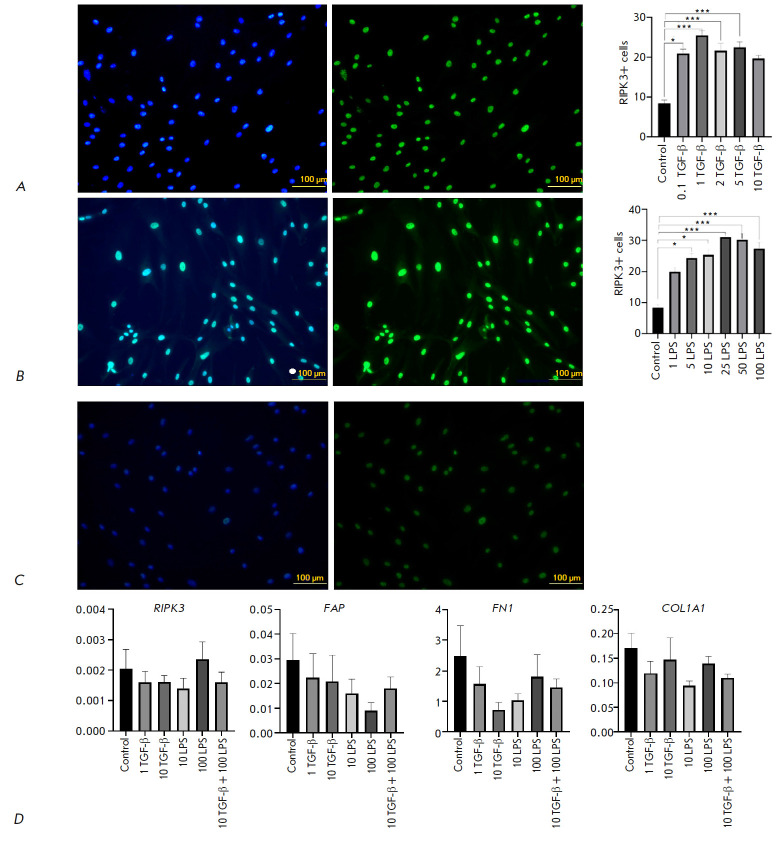
Expression patterns of RIPK3 and ECM synthesis markers in human dermal fibroblasts. Human dermal fibroblasts cultured in medium containing TGF-β (*A*), LPS (*B*) and untreated (*C*), stained with antibodies to RIPK3, nuclei stained with DAPI, 20× magnification (left) scale bars 100 μm; statistical analysis of fluorescence intensity using the Kruskal–Wallis test, *P < 0.05, ***P < 0.001 the fluorescence intensity data are presented as averages with a spread in the form of an average error (right). Expression of ECM synthesis markers and *RIPK3 *in the presence of TGF-β and LPS in cultured human dermal fibroblasts, RT-PCR *P *> 0.05 (Mann–Whitney U test, gene expression data are presented as average values with a spread in the form of an average error (*D*)

An analysis of human dermal fibroblasts stained with antibodies to RIPK3 showed that addition of TGF-β at concentrations of 0.1, 1, 2, and 5 ng/ml (*Fig. 3A*) and LPS at concentrations 5, 10, 25, 50, and 100 ng/ml (*Fig. 3B*) results in a reliable increase in the fluorescence intensity. This indicates that RIPK3 expression can be regulated by TGF-β1 and/or TLR4/NF-κB signals. However, a comparison of real- time PCR results for *RIPK3 *did not reveal significant differences between the control and analyzed cells (*Fig. 3D*). Real-time PCR analysis of markers of activated fibroblasts, namely *FAP, FN1, *and *COL1A1*, did not show significant differences between the experimental groups and the control. This result can be also due to the change in the cell phenotype in a 2D culture. The fibroblast phenotype is known to change depending on the substrate. Culturing of mouse lung fibroblasts in hydrogels with differing stiffness can lead to different cell phenotypes: with high expression levels of α-SMA (α-SMA Hi) and FAP (FAP Hi). A direct correlation of gene expression with the substrate stiffness is observed in α-SMA Hi, while a reverse correlation is noted in FAP Hi [22]. In addition, our study was performed in human and mouse primary dermal fibroblasts, which differ from the cells used in the studies with the methodology and concept we relied on. The study by Imamura was performed using NIH 3T3 mouse embryo fibroblasts and human kidney fibroblasts; Guo et al. used human uterine fibroid cells [5, 8]. Thus, the *in vitro* model of fibrosis may not be the most suitable for studying the activation of human dermal fibroblasts and the role of RIPK3 in it. It is necessary to develop another *in vitro *model to better grasp the role of RIPK3 in wound healing. Fibroblast cultures in collagen gel or organoids that preserve epithelial-mesenchymal interactions may be a promising approach in solving this riddle.

## CONCLUSION

A bioinformatics analysis of the data showed that human keloid scar tissue contains significantly more RIPK3+ fibroblasts compared to normal skin. RIPK3+Vim+ cells were found both in mouse wound bed and human keloid. The number of Vimentin+RIPK3+ cells during skin regeneration in mice was significantly higher compared to that in normal dermis. The expression of the *Ripk3 *and ECM synthesis markers *Acta2*, *Fap*, *Col1a1, *and *Fn1 *in cells isolated from a mouse wound bed indicates that these cells are fibroblasts. The fluorescence intensity was significantly higher after staining with antibodies to human RIPK3 fibroblasts treated with LPS at concentrations of 5, 10, 25, 50, and 100 ng/ml and TGF-β at concentrations of 0.1, 1, 2, and 5 ng/ml compared to the control. Real-time PCR revealed no significant differences in the expression level of the ECM synthesis genes *FAP*, *FN1*, *COL1A1*, and *RIPK3 *between human dermal fibroblasts treated with these substances and the control. This result is controversial and requires further research. It is possible that RIPK3 expression in wound fibroblasts is not directly associated with fibrotic processes, while RIPK3 plays another, yet unknown, role in wound healing.

## Abbreviations

RT-PCR: real-time polymerase chain reaction
ECM: extracellular matrix
RIPK3: receptor- interacting serine/threonine-protein kinase 3
PFA: paraformaldehyde
DEG: differentially expressed gene
Vim: Vimentin
LPS: lipopolysaccharide
Fn: fibronectin
FAP: fibroblast activation protein-α
Col1a1: collagen type I alpha 1
UMAP: Uniform Manifold Approximation and Projection
